# Does the Extreme Male Brain Hypothesis of Autism Apply More to Females Than Males? A Systematic and Meta‐Analytic Approach

**DOI:** 10.1002/aur.70198

**Published:** 2026-02-16

**Authors:** Cory Szakal, Bernard Crespi

**Affiliations:** ^1^ Department of Biological Sciences Simon Fraser University, 8888 University Drive Burnaby British Columbia Canada

**Keywords:** autism, empathizing, extreme male brain, prenatal testosterone, systemizing

## Abstract

The extreme male brain (EMB) hypothesis posits that autism risk is mediated by high systemizing and low empathizing. This hypothesis has accrued extensive support, but the degree to which it applies in females compared to males, and the relative extent to which autism is associated with empathizing compared to systemizing, is unclear. Systematic review and meta‐analyses of studies measuring the empathy quotient (EQ), the systemizing quotient (SQ), and the autism quotient (AQ), among individuals with autism and neurotypical individuals, were used to address these questions. Analyses of results from 34 studies indicated that: (1) Females show larger proportional differences in EQ and SQ between ASD and NT individuals than do males, (2) EQ shows larger proportional differences between autism spectrum (ASD) and neurotypical (NT) individuals than does SQ, (3) sex differences in EQ and SQ are highly attenuated among individuals with ASD, especially for SQ in females, (4) the regressions of EQ and SQ on AQ show significantly steeper slopes among individuals with ASD than in NT individuals, and (5) across studies, EQ and SQ are inversely associated among individuals with ASD, but not in NT individuals. These results provide new insights into the causes of ASD and its male bias.

## Executive Summary

The ‘extreme male brain’ theory of autism suggests that autism represents an exaggeration of two male‐typical traits, relatively low empathy (‘empathizing’) and relatively high interest in non‐living systems like engineering or classification (‘systemizing’), and that high testosterone in the womb contributes to this pattern. This study reviewed available evidence on levels of empathizing and systemizing in relation to autism among males and females, to see if these two traits had similar effects in the two sexes. The results showed that shifts in empathizing and systemizing were much greater in females than males, which can help to explain the male‐biased sex ratio in autism.

## 1 Introduction

One of the most prevalent findings in autism spectrum disorder (ASD) research is an increased prevalence in natal (assigned at birth) males, with approximately four males diagnosed for every one female overall (Ferri et al. 2018). Sex differences are more pronounced among individuals with higher intellectual ability, with males substantially overrepresented among individuals with average to high intelligence, while males and females are more equally represented among individuals with intellectual disability (Werling and Geschwind 2013).

Several nonexclusive hypotheses have been proposed to help explain sex differences in ASD diagnoses. First, autistic females may present with the same core traits but with different manifestations of these traits, leading to underdiagnosis and misdiagnosis (e.g., Kopp and Gillberg 1992; Lai et al. 2022; Cardon et al. 2023). For example, autistic males tend to exhibit more externalizing behavior problems (e.g., aggression, hyperactivity) and more overt repetitive behaviors and restricted interests, whereas females tend to display more internalizing behaviors (e.g., anxiety, depression) and demand avoidance, along with subtler repetitive behaviors, heightened attention to socially salient stimuli, and social‐communication styles that can appear more reciprocal or socially attuned (Cardon et al. 2023; Lai et al. 2022). Male‐typical presentations of autism may thus be more likely to prompt ASD evaluations, whereas female‐typical manifestations may be noticed only upon assessment of intellectual disability (Werling and Geschwind 2013). The fact that historically developed diagnostic criteria have been based on male presentations may also contribute to sex‐associated diagnostic discrepancies; females may have the same likelihood of developing the disorder but are not being identified to the same degree (De Giambattista et al. 2021; Dworzynski et al. 2012).

A second hypothesis proposed to explain sex differences in autism diagnosis is centered around camouflaging, which represents the act of masking, compensating for, or attempting to conceal autistic traits (e.g., forcing eye contact, imitating social movements, scripting conversations; Cook et al. 2021). Autistic females tend to camouflage more frequently and intensely than autistic males (Cook et al. 2021; Cruz et al. 2025), which is thought to arise in part from societal pressures and expectations (e.g., gendered norms emphasizing empathy, social reciprocity, politeness, and emotional expressiveness in girls) that elevate their motivation to fit in and hide autistic traits (Klein et al. 2025; Tien et al. 2025). Camouflaging not only reduces the visibility of autistic traits, but it can also carry significant psychological costs (e.g., burnout, exhaustion, depression, anxiety) that may contribute to misdiagnosis (Hull and Mandy 2017). Thus, females may have similar severities as males but the probability of ascertainment is reduced because they are better able to conceal their autistic phenotypes (De Giambattista et al. 2021).

A third explanation for higher diagnosis rate in males than females posits that certain biological factors make females more resistant to ASD, such that a higher genetic, phenotypic and/or environmental ‘load’ is required for a diagnostic‐threshold level of traits to manifest in females compared to males. This hypothesis is supported by genetic evidence, in that higher mutational ‘burdens’ across autism‐associated rare and common variant classes have been observed in autistic females (Antaki et al. 2022; Jacquemont et al. 2014; Warrier et al. 2022; Wigdor et al. 2022; Zhang et al. 2020), whose family members also display elevated genetic liability and odds of ASD diagnosis (Jacquemont et al. 2014; Wigdor et al. 2022), suggesting that a greater genetic load may be required for females to be diagnosed. Additionally, in two large twin samples, siblings of autistic females had more autistic traits and higher recurrence risk than siblings of autistic males, supporting the theory; thus, if females require a greater biological and/or genetic load to manifest autism, then their relatives should carry more subclinical traits (Robinson et al. 2013). Some developmental factors may therefore confer protection against ASD in females, implying that something inherent in their biology reduces the likelihood of diagnosis. If this hypothesis is correct, then females would be expected to show greater shifts in phenotypic, as well as genetic, causes of autism, since they require a greater perturbation from typical phenotypes to reach diagnostic thresholds.

Conceptual frameworks that consider sex‐based differences in biological and psychological developmental trajectories are central to understanding the male‐biased prevalence of ASD. One influential framework that integrates cognitive psychological style with biological influences is Baron‐Cohen's Empathizing–Systemizing theory of sex differences, whereby neurotypical (NT) females tend toward higher empathizing, whereas NT males tend toward higher systemizing, as measured via the Empathy Quotient (EQ) and Systemizing Quotient (SQ), respectively (Baron‐Cohen et al. 2002, 2011). According to the Extreme Male Brain (EMB) hypothesis, autistic traits thus reflect extreme development of male‐typical cognitive profiles, as evidenced by higher scores on the SQ combined with lower scores on the EQ compared to neurotypical populations, with higher fetal testosterone (FT) proposed as a primary biological mechanism (Baron‐Cohen 2002). This conceptualization provides a potential physiological and psychological explanation for the consistent male bias in ASD, as males are exposed to higher levels of FT (Baron‐Cohen et al. 2011). The EMB hypothesis is notably supported by population studies, which demonstrate the expected shifts in EQ and SQ among ASD individuals compared to NT, with reductions in typical sex differences in ASD groups (Baron‐Cohen et al. 2014; Greenberg et al. 2018). Such attenuation of psychological sex differentiation led Baron‐Cohen et al. (2014) to suggest a “more pronounced effect of ‘masculinization’ (i.e., shifting towards the typical male‐end of the profile) in females with autism.”

Although a large body of work supports the EMB hypothesis, systematic reviews or meta‐analyses that collectively quantify patterns of sex differences in empathizing and systemizing across autistic and NT populations have yet to be conducted. In a foundational neuroimaging study, Lai et al. (2013) demonstrated that ASD interacts with sex in ways that alter the magnitude and pattern of ‘masculinization’, with autistic females and males showing partially distinct, sex‐moderated patterns of ‘brain‐type’ organization: autistic females in particular exhibit shifts toward more male‐distinct profiles, with neuroanatomical expressions that differed from those of autistic males. Such sex‐moderated neural profiles may correspond to differences in empathizing and systemizing, and evaluating these traits without stratifying by sex risks obscuring sex‐specific pathways, highlighting the need for sex‐stratified analyses to test EMB predictions. Addressing this gap may help to clarify the degree to which cognitive profiles align with EMB theory predictions in each sex, and elucidate why rates of autism diagnosis differ between females and males.

This study systematically evaluates the extent to which the EMB theory is supported by data from autistic and NT males and females, by quantifying sex differences in empathizing and systemizing across these groups using all relevant data published to date. According to the EMB framework, both autistic males and autistic females are expected to exhibit lower EQ scores and higher SQ scores compared to their NT counterparts. However, the relative magnitudes of these cognitive profile shifts may differ between females and males, and between EQ and SQ; for example, the contrasts in EQ and SQ between autistic and NT females may differ from those between autistic and NT males. By directly comparing EQ, SQ, and Autism Quotient (AQ) profiles between autistic and NT males and females, this study tests predictions of the EMB theory while seeking to clarify how sex may modulate the expression of autism in the two sexes. The primary specific question addressed is thus the degree to which, across studies, sex differences in EQ and SQ vary between individuals diagnosed with ASD and controls. These data also allow evaluation of the relative degree to which variation in EQ and SQ contribute to autism diagnoses and AQ and provide insights into the question of how variation in EQ and SQ may contribute to the pronounced sex bias in autism.

## 2 Methods

### 2.1 Systematic literature searches

A systematic literature search was conducted using PubMed, Web of Science, and PsycINFO to identify studies assessing empathizing and systemizing in autistic and NT populations, as outlined in Table S1 and in Figure S1. Searches were conducted between January and February 2025 following PRISMA‐informed procedures, and results were limited to peer‐reviewed journal articles that analyzed human participants and utilized quantitative methodologies. Duplicates were removed, and titles and abstracts were screened for relevance by a single reviewer. Studies were included if they (1) reported EQ and/or SQ scores (2) included autistic participants or included NT individuals who completed the Autism Quotient (AQ), and (3) provided effect sizes or sufficient data for effect size extraction (e.g., means, standard deviations, and sample sizes). Full‐text screening was conducted for all eligible articles, and final inclusion was determined based on the availability of the required sex‐stratified cognitive trait data for both autistic and neurotypical groups. A formal risk‐of‐bias assessment was not conducted, given that the included studies relied on standardized, self‐report cognitive measures with consistent scoring procedures. Potential bias was indirectly addressed via strict inclusion/exclusion criteria and extraction of only clearly reported quantitative data.

### 2.2 Statistical analyses

Different versions of the AQ, EQ, and SQ were used by different studies. To make these measures comparable quantitatively, means were divided by the maximum possible score for each questionnaire, making all of the values fall between zero and 100% for analyses.

All statistical analyses were conducted in R 4.2.2. The rma() function from the metafor package was used to fit a meta‐analytic random‐effects model, which accounts for variability in true effect sizes between studies by incorporating both within‐study and between‐study variance. This approach is used when there is significant heterogeneity, which is expected in this case considering the diversity of participants assessed and differences in sample characteristics and measurement tools.

To examine how EQ and SQ scores relate to autistic traits and whether scores differ by sex, meta‐regression models were utilized with AQ scores as the dependent variable and either EQ or SQ scores, sex, and their interaction (e.g., EQ*sex, SQ*sex) as moderators. Covariates such as age were not included because age data was inconsistently reported across studies, often aggregated across sexes or not available in a form permitting diagnosis or sex‐stratified extraction. Models were fitted using the rma() function from the metafor R package, with restricted maximum likelihood (REML) estimation, and sampling variances were used to weight studies.

## 3 Results

By systematic review, a total of 34 studies comprising 1,234,560 total participants (757,726 females and 476,834 males) were eligible for inclusion in this study (Tables [Link], [Link], Figure S1). Descriptive data for EQ, SQ, and AQ demonstrate the expected patterns related to sex and diagnosis, with higher NT female than male average values for EQ, higher NT male than female average values for SQ, lower EQ among autistic females and males compared to same‐sex NT, and higher SQ among autistic females and males compared to same‐sex NT (descriptive statistics in Table 1, Cohen's d scores in Table 2). The SQ‐EQ Bias score (also called the D‐score), which quantifies the combined linear effects of EQ and SQ, also showed the pattern expected under the EMB hypothesis (Table 2).

**TABLE 1:** Means and standard deviations for EQ, SQ, and AQ for each group, presented as the reported mean divided by the maximum score for the quotient version used multiplied by one hundred. *N* = number of studies.

	EQ	SQ	AQ
	(% maximal score)		
	Mean (SD) (N)		
M_ASD_	26.3 (13.2) (23)	46.3 (17.4) (18)	62.6 (15.5) (11)
F_ASD_	30.7 (14.6) (22)	45.5 (17.5) (17)	60.0 (15.8) (11)
M_NT_	52.1 (15.1) (31)	42.8 (15.8) (28)	36.3 (14.5) (22)
F_NT_	60.6 (14.5) (31)	34.8 (13.8) (28)	32.9 (13.7) (22)

**TABLE 2:** Random‐effects meta‐analytic results for (A) between‐group differences in EQ and SQ between autistic and NT males (M) and females (F), and (B) within‐group SQ‐EQ bias scores, representing the difference between SQ and EQ within each diagnostic‐sex group.

(A)		EQ	
		Pooled Cohen's d	
	df	d (95% CI)	SE
M_ASD_—M_NT_	18	−1.48 (−1.75,‐1.21)***	0.137
F_ASD_—F_NT_	17	−1.87 (−2.24,‐1.49)***	0.193
M_ASD_—F_ASD_	21	−0.30 (−0.41,‐0.19)***	0.055
M_NT_—F_NT_	30	−0.61 (−0.71,‐0.52)***	0.048
		**SQ**	
		Pooled Cohen's d	
	df	d (95% CI)	SE
M_ASD_—M_NT_	15	0.32 (0.16.0.48)***	0.083
F_ASD_—F_NT_	14	0.68 (0.43, 0.92)***	0.125
M_ASD_—F_ASD_	16	0.14 (0.03, 0.29)*	0.065
M_NT_—F_NT_	27	0.55 (0.43, 0.66)***	0.057
(B)		**SQ**—**EQ (Bias score)**	
		Pooled Cohen's d	
	df	d (95% CI)	SE
M_ASD_	17	1.08 (0.74, 1.41)***	0.171
F_ASD_	16	0.73 (0.32, 1.14)***	0.222
M_NT_	27	−0.56 (−0.71, −0.41)***	0.078
F_NT_	27	−1.76 (−2.00, −1.53)***	0.115

* 0.01 < *p*‐value < 0.05.

** 0.001 < *p*‐value < 0.01.

*** *p*‐value ≤ 0.001.

**FIGURE 1:**
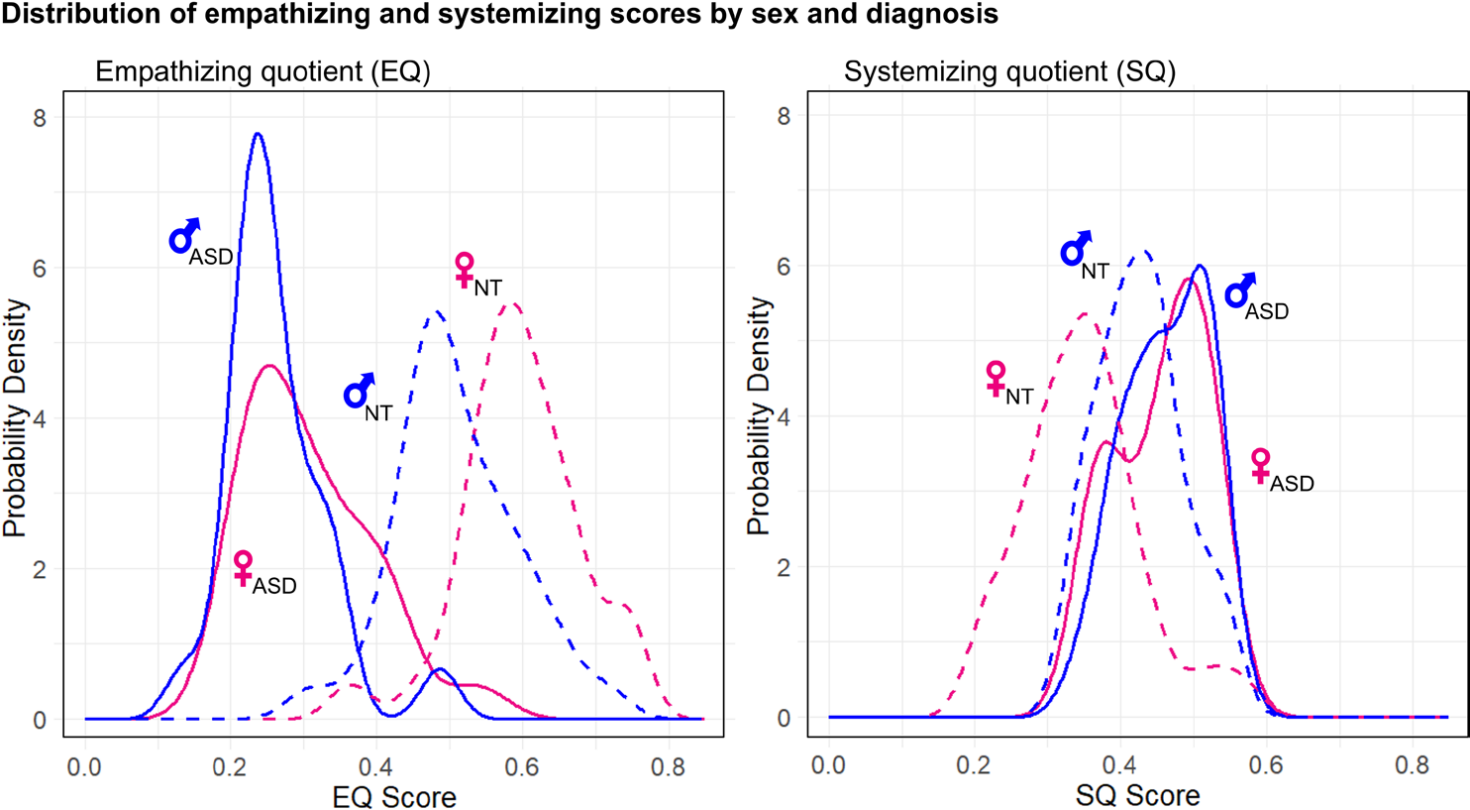
Distributions of EQ scores (left) and SQ scores (right) in each group. Lines are estimated via kernel density estimation, with cumulative distribution function plots on the right to provide a clearer distinction between groups. Solid lines represent autistic groups; dashed lines represent neurotypical groups. Males and females are colored blue and red, respectively.

**FIGURE 2:**
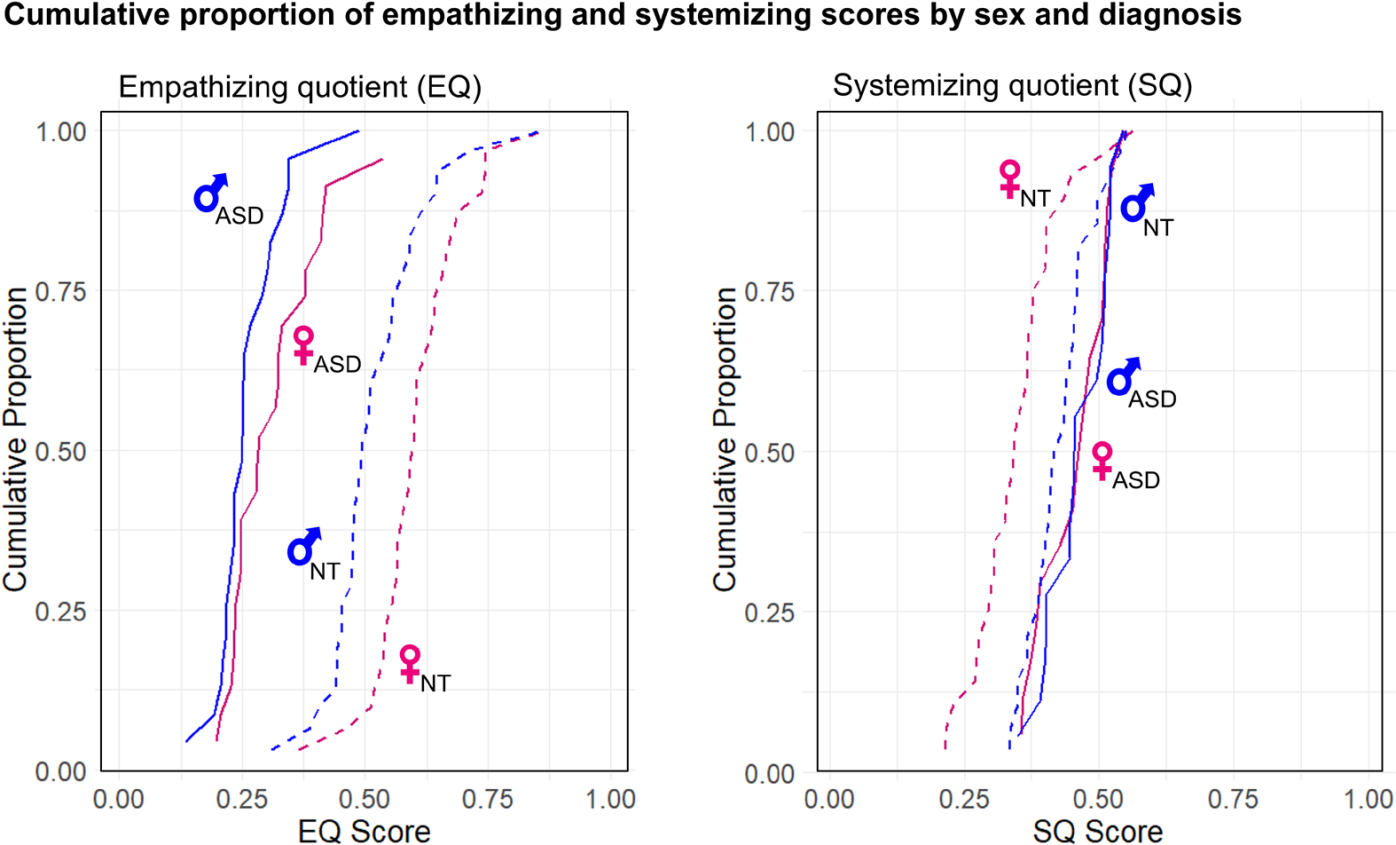
Cumulative distributions of EQ scores (left) and SQ scores (right) in each group. Solid lines represent autistic groups; dashed lines represent neurotypical groups. Males and females are colored blue and red, respectively.

The density and frequency distributions of EQ and SQ in relation to the four diagnosis and sex groups provide further information concerning how empathizing and systemizing relate to thresholds for ASD diagnoses (Figures 1 and 2). Three main patterns are evident from analyses of the data that generate these distributions. First, the differences in EQ and SQ between ASD and NT individuals are significantly larger in females than in males (paired *t*‐tests, EQ: t = 2.61, *p* = 0.018, df = 17, Cohen's d = 0.28; SQ: t = 2.47, *p* = 0.027, df = 14, Cohen's d = 0.29). These results indicate that ASD‐NT group differences in EQ and SQ are greater in females than in males.

Second, the percent differences between ASD and NT individuals are substantially and significantly larger for EQ than for SQ, for both females (EQ difference 29.8, SD = 14; SQ difference 9.8, SD = 8, df = 14 for both; paired *t*‐test, t = 5.49, *p* < 0.001) and males (EQ difference 25.4, SD = 15; SQ difference 4.7, SD = 5, df = 15 for both; paired *t*‐test, t = 5.49, *p* < 0.001). These findings suggest an increased sensitivity of the EQ in diagnosing ASD on a proportional basis, as regards these two questionnaires.

Third, as noted by Baron‐Cohen et al. (2014), sex differences in EQ and SQ show evidence of being attenuated among individuals with ASD (Table 3). SQ in particular showed no significant difference, and a very small effect size (0.14), upon comparing autistic females to autistic males by paired *t*‐test (see Tables 2 and 3). In comparison, EQ showed a highly significant difference (*p* < 0.001), with an effect size about twice as large (0.30) as that for SQ, for the comparison of autistic females and autistic males.

**TABLE 3:** Results from paired t‐tests comparing autistic and NT males (M) and females (F) for differences in EQ, SQ and D (BIAS) scores.

	EQ SCORES			SQ SCORES			BIAS (SQ‐EQ) SCORES		
	Mean difference (95% CI)	Paired t‐Value	df	Mean difference (95% CI)	Paired t‐Value	df	Mean difference (95% CI)	Paired t‐Value	df
M_ASD_—M_NT_	−0.25 (−0.32, −0.19)	−8.16***	18	0.05 (0.02, 0.07)	3.77**	15	0.28 (0.21, 0.35)	8.61***	15
F_ASD_—F_NT_	−0.30 (−0.37, −0.23)	−8.98***	17	0.10 (0.05, 0.14)	4.59***	14	0.38 (0.30, 0.47)	9.56***	14
M_ASD_—F_ASD_	−0.05 (−0.07, −0.03)	−4.82***	21	0.01 (−0.01, 0.03)	1.20	16	0.06 (0.02, 0.09)	3.05**	16
M_NT_—F_NT_	−0.09 (−0.10, −0.07)	−12.4***	30	0.08 (0.06, 0.10)	8.24***	27	0.17 (0.14, 0.19)	14.1***	27

* 0.01 < *p*‐value < 0.05.

** 0.001 < *p*‐value < 0.01.

*** *p*‐value ≤ 0.001.

**FIGURE 3:**
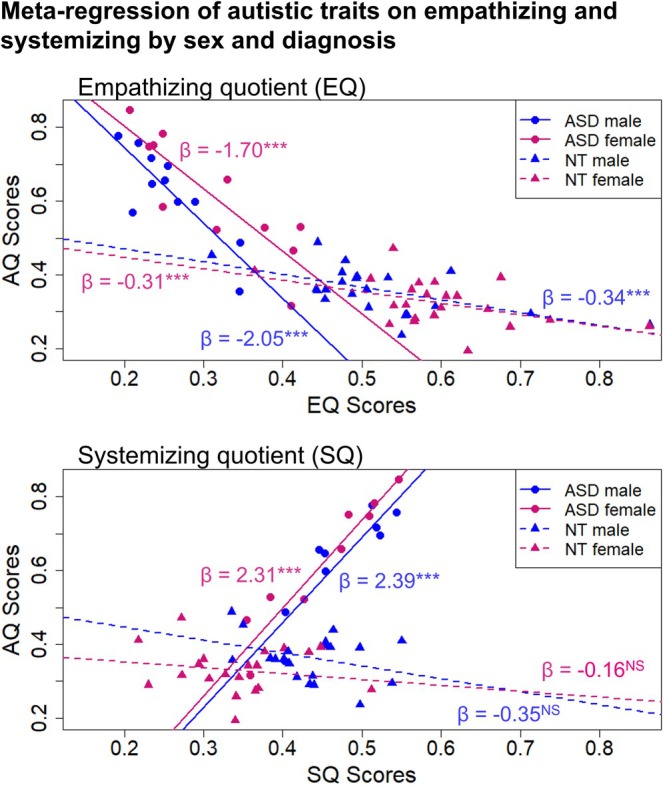
Meta‐regressions of AQ on EQ or SQ, in relation to sex and diagnostic status. *: 0.01 < *p* value < 0.05, **: 0.001 < *p*‐value < 0.01, ***: *p*‐value ≤ 0.001.

In the context of these results, how are empathizing and systemizing related to autism quotient scores, among NT and ASD individuals? Meta‐regressions (Figure 3) indicate that AQ was significantly inversely related to EQ among ASD and NT females and males. By contrast, AQ was significantly positively related to SQ among autistic females and males, but AQ is not significantly related to SQ among NT females or males. The slopes of the relationships of AQ with EQ and SQ were also significantly steeper for autistic females and males compared to NT individuals (Table 4, interaction terms all *p* < 0.001). These findings indicate that, in these data, associations of EQ and SQ with AQ are stronger among ASD individuals than among NT individuals.

**TABLE 4:** Results from meta‐analytic regression models evaluating the effects of EQ and SQ on AQ among females and males. Significant slope differences (see Figure 3) are indicated by the interaction terms.

	AQ ~ EQ*DIAGNOSIS			
	df		β (SE)	Adjusted R^2^
Females	29	EQ	−1.71 (0.268)***	0.846
		Diagnosis	−0.64 (0.128)***	
		Interaction	1.40 (0.311)***	
Males	29	EQ	−2.05 (0.364)***	0.856
		Diagnosis	−0.62 (0.115)***	
		Interaction	1.71 (0.382)***	
	AQ ~ SQ*DIAGNOSIS			
	df		β (SE)	Adjusted R^2^
Females	25	SQ	2.39 (0.313)***	0.874
		Diagnosis	0.84 (0.160)***	
		Interaction	−2.55 (0.373)***	
Males	25	SQ	2.31 (0.410)***	0.837
		Diagnosis	0.98 (0.221)***	
		Interaction	−2.64 (0.475)***	

* 0.01 < *p*‐value < 0.05.

** 0.001 < *p*‐value < 0.01.

*** *p*‐value ≤ 0.001.

**FIGURE 4:**
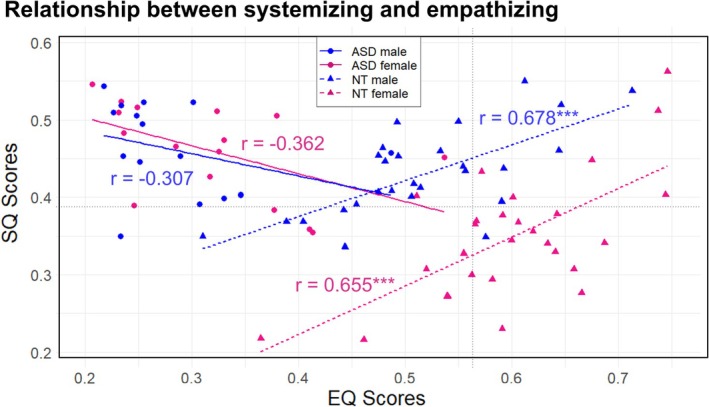
Correlations of systemizing with empathizing, in relation to sex and diagnostic status. *: 0.01 < *p*‐value < 0.05, **: 0.001 < *p*‐value < 0.01, ***: *p*‐value ≤ 0.001.

The ASD and NT groups also differed in the statistical relationships of EQ with SQ. Thus, in the NT group, EQ and SQ were significantly positively related to one another, but in the ASD group of females combined with males, these two variables were inversely related (Figure 4). Combining the ASD and NT groups together, under a model considering ASD as dimensional, results in U‐shaped relationships for both males and females (*p* < 0.001 for quadratic term in each, overall R^2^ = 0.25 in males and 0.36 in females) (Figure S2). The NT group also shows the expected mean differences between females and males in EQ and SQ. By contrast, the ASD group shows an absence of bivariate sexual differences, such that the two regression lines virtually overlap.

## 4 Discussion

The systemizing‐empathizing theory for autism is strongly supported by the meta‐analytic results reported here. Within this broad context, several main findings characterize the relationships between EQ, SQ, AQ, and autism diagnoses in females compared to males.

First, the EQ shows a larger proportional difference (3 to 5‐fold larger) between ASD and NT individuals than does SQ, in both females and males. To the extent that unit changes in EQ and SQ can be considered equivalent in their effects on ASD risk, these findings suggest that empathizing may have larger effects overall than systemizing in contributing to the psychological differences that characterize autism diagnoses.

Second, the differences in females between ASD and NT individuals for EQ and SQ are significantly larger than the differences in males. The EMB hypothesis can thus be considered to apply more strongly to females than to males. Given EQ and SQ as psychological causes of variation in AQ and the likelihood of ASD diagnosis, this pattern supports the hypothesis that greater phenotypic shifts in these two traits may be necessary for females than for males to result in a diagnosis of ASD. Because EQ and SQ are heritable, such shifts are expected to be underlain by genetic and epigenetic factors. These results for EQ and SQ are concordant with results showing that a larger genetic ‘loading’ for ASD traits is necessary for females, than for males, to result in a diagnosis (Jacquemont et al. 2014; Wigdor et al. 2022; Zhang et al. 2020), with females also exhibiting stronger ASD‐associated methylation signatures compared to males (Mouat et al. 2025). As such, the apparent requirement for larger shifts in EQ and SQ among females than males, to result in an autism diagnosis, may contribute to the male bias in the prevalence of ASD. The increased size of such shifts in females, and effects of female protection from autism more generally, may be related to the fact that effects from increases in prenatal and postnatal testosterone would be expected to be more deleterious to female than to male fitness, given that relatively high testosterone has negative effects on female development and reproduction but generally positive effects in males (Lidborg et al. 2022; Crespi et al. 2025).

Third, the substantial and significant differences in EQ and SQ between NT males and NT females contrast strikingly with the small and, for SQ, non‐significant differences between autistic males and autistic females. ASD individuals thus show clear evidence for attenuation of typical psychological sex differences in these traits, especially for SQ. This attenuation may partly reflect gendered social expectations: autistic traits in females are often reframed as shyness, sensitivity, or conscientiousness, and ASD females may camouflage low‐EQ or high‐SQ traits to align with female‐related norms, whereas ASD males experience less pressure to mask systemizing strengths or low levels of empathy (Tien et al. 2025), contributing to greater phenotypic similarities. An apparent requirement for more‐substantial upward shifts to male‐typical systemizing values, for females to develop ASD, may also promote the male bias in autism, given that such shifts involve increases and enhancements of psychological traits, which are expected to occur less readily (via genetic and environmental effects) than are decreases and dysregulation leading to deficits. The attenuation or absence of psychological sex differences in EQ and SQ among individuals with ASD also supports the hypothesis that autism etiology involves effects of factors directly underlying sex differences per se, as expected for effects of prenatal testosterone (Baron‐Cohen et al. 2011).

Differences between ASD and NT individuals are also evident for the relationships of EQ with SQ, which is negative among individuals diagnosed with ASD, but positive in NT males and NT females, yielding a U‐shaped pattern if the two groups are pooled. A comparable directional difference in EQ‐SQ correlations between ASD individuals (r = −0.29) and NT individuals (r = −0.09) was found by Wheelwright et al. (2006). Despite the greater sensitivity of the EQ, the opposite EQ‐SQ associations in ASD individuals indicate that the SQ still provides distinct clinical information, such that elevated systemizing may reflect alternative cognitive routes or compensatory strategies not captured by EQ alone. Together, these findings are suggestive of a cognitive trade‐off between empathizing and systemizing in individuals diagnosed with ASD, but not, or less so, in NT individuals. If such a negative correlation has a causal basis, then increases in SQ should be associated with decreases in EQ and vice versa, with both sets of changes resulting in higher scores for the autism quotient AQ. Moreover, changes in EQ and SQ would be associated with greater effects on AQ score among autistic individuals than in NT individuals, given the steeper slopes for the regressions of AQ on SQ and AQ on EQ among individuals with ASD, compared to NT.

Trade‐offs are caused by forms of incompatibility between the expression of two or more beneficial traits. The causes of any psychological trade‐offs between empathizing and systemizing remain unknown, but in non‐cognitive contexts, trade‐offs are due to physical or physiological incompatibilities that typically are expressed, or intensified, when some relevant resource is limiting or constrained (Van Noordwijk and De Jong 1986). Cognitive trade‐offs, as evidenced for example by negative correlations between mathematical ability and empathy (e. g, Escovar et al. 2016), may thus involve specializations driven by partial incompatibilities between different mental traits or abilities (Crespi 2015; Del Giudice and Crespi 2018). In the context of ASD, a hypothesis based on such trade‐offs may involve increased systemizing interfering with empathizing, by generating a cognitive style that centers on non‐social, rule‐based stimuli, or by a systemizing cognitive style being associated with hyper‐focused non‐social attention (Baron‐Cohen et al. 2009). By this hypothesis, ASD individuals mostly rely on analytical neural pathways during social tasks whereas NT individuals preferentially recruit social‐affective circuits (Di Martino et al. 2009; Lombardo et al. 2010). Alternatively, sensory hypersensitivities may result in increased systemizing, reduced empathizing, or both (e.g., Tavassoli et al. 2018; Smees et al. 2024). The hypothesis is supported by the findings that scores on the Sensory Perception Quotient show a strong, significant positive correlation with SQ (*r* = 0.47), and a significant negative correlation with EQ (r = −0.15) (Greenberg et al. 2018). It remains unclear why any such trade‐off would manifest only in some individuals or groups (those with diagnoses of ASD) or generate strong negative correlations between EQ and SQ only in some populations and studies. Given that inverse causal relationships between empathizing and systemizing would be expected to directly promote the low‐EQ, high‐SQ combination that typifies autism under the EMB model, these findings should motivate studies on where, when and how cognitive trade‐offs involving empathizing, systemizing, and ASD develop and are expressed. They may be key to psychological aspects of ASD.

Further studies of the EMB and sex differences in autism prevalence can usefully focus on the causes of between‐study variation in EQ, SQ and AQ among ASD individuals in comparison to controls. In this context, the findings of substantially different relationships of AQ with EQ and SQ, and of EQ with SQ, between ASD and NT individuals suggest differences in cognitive structure between these two groups, that may be related to variation in the presence and strength of trade‐offs between aspects of social and non‐social cognition (Crespi 2015). Given the notably reduced sex difference in SQ for ASD individuals, the results reported here also suggest a distinct role for increased systemizing among autistic females, that may also help to explain sex differences in the prevalence of this condition. Additional studies of the causes and effects of variation in systemizing among females with and without ASD are thus especially motivated by the results reported here. Because a substantial portion of the total sample size, and thus much of the statistical power, derives from studies conducted by a small number of research groups, future work should also evaluate whether such clustering in data collection contributes to the between‐study patterns observed.

## Funding

This work was supported by a Canada Research Chair to BJC.

## Conflicts of Interest

The authors declare no conflicts of interest.

## Supporting information


**Figure S1:** aur70198‐sup‐0001‐FigureS1.tif.

**Figure S2:** aur70198‐sup‐0002‐FigureS2.tif.

**Table S1:** Articles obtained through database searches. Keywords were run through PubMed (Pmed), Web of Science (WoS), and PSYCInfo (PI), and the results were recorded (results column). Each database allowed for the use of different filters: in Pmed, searches were restricted to studies in English, humans were selected as the species, and quantitative methodologies were chosen (adaptive clinical trial, classical article, clinical study, clinical trial, clinical trial phase I‐IV, comparative study, controlled clinical trial, dataset, meta‐analysis, pragmatic clinical trial, randomized control trial, twin study, validation study). In PI, searches were restricted to studies in English, humans were selected as species, and quantitative methodologies were chosen (brain imaging, empirical study, quantitative study, interview, experimental replication, clinical case study, follow‐up study, longitudinal study, meta‐analysis, twin study).

**Table S2:** Mean and SD of EQ and SQ scores in autistic and NT males and females, presented as the reported mean divided by the maximum score for the quotient version used multiplied by one hundred for comparison.

**Table S3:** Mean and standard deviations of AQ, EQ and SQ scores in NT males and females, presented as the reported mean divided by the maximum score for the questionnaire version used multiplied by one hundred for comparison.

## Data Availability

The data that supports the findings of this study are available in the Supporting Information of this article.
