# Supplementary figures and images for: Does the Extreme Male Brain Hypothesis of Autism Apply More to Females Than Males? A Systematic and Meta‐Analytic Approach
Source: Autism Res. 2026 Feb 16;19(4):e70198. doi: 10.1002/aur.70198 (PMC13087839; doi:10.1002/aur.70198)

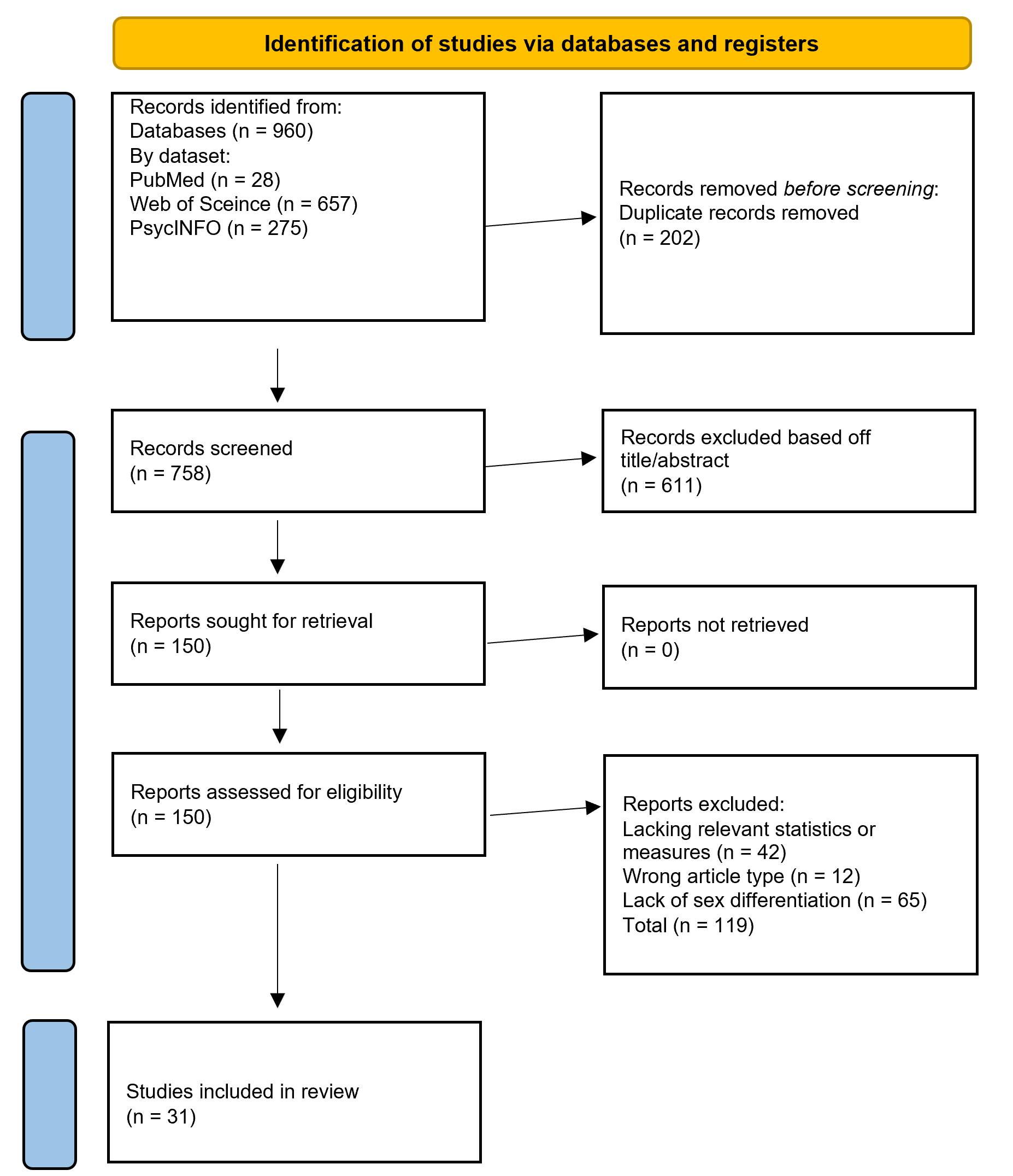

Supplement: Supplementary file 1 — Figure S1: aur70198‐sup‐0001‐FigureS1.tif. [file AUR-19-0-s005.tif]

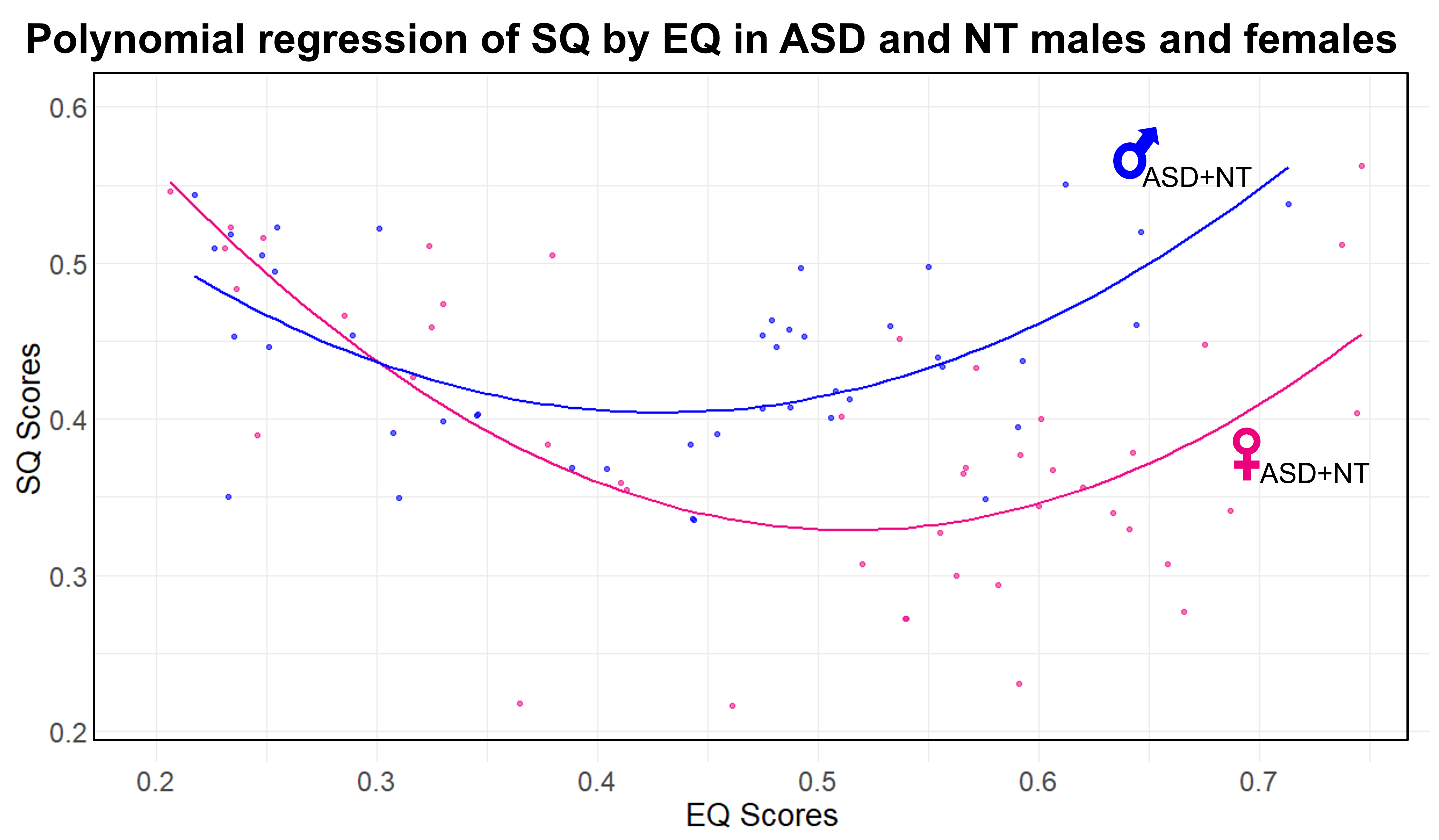

Supplement: Supplementary file 2 — Figure S2: aur70198‐sup‐0002‐FigureS2.tif. [file AUR-19-0-s002.tif]
