# Supplementary material for: Does the Extreme Male Brain Hypothesis of Autism Apply More to Females Than Males? A Systematic and Meta‐Analytic Approach
Source: Autism Res. 2026 Feb 16;19(4):e70198. doi: 10.1002/aur.70198 (PMC13087839; doi:10.1002/aur.70198)
Supplement: Supplementary file 3 — Table S1: Articles obtained through database searches. Keywords were run through PubMed (Pmed), Web of Science (WoS), and PSYCInfo (PI), and the results were recorded (results column). Each database allowed for the use of different filters: in Pmed, searches were restricted to studies in English, humans were selected as the species, and quantitative methodologies were chosen (adaptive clinical trial, classical article, clinical study, clinical trial, clinical trial phase I‐IV, comparative study, controlled clinical trial, dataset, meta‐analysis, pragmatic clinical trial, randomized control trial, twin study, validation study). In PI, searches were restricted to studies in English, humans were selected as species, and quantitative methodologies were chosen (brain imaging, empirical study, quantitative study, interview, experimental replication, clinical case study, follow‐up study, longitudinal study, meta‐analysis, twin study). [file AUR-19-0-s004.docx]

**Supplementary table 1.** Articles obtained through database searches. Keywords were run through PubMed (Pmed), Web of Science (WoS), and PSYCInfo (PI), and the results were recorded (results column). Each database allowed for the use of different filters: in Pmed, searches were restricted to studies in English, humans were selected as the species, and quantitative methodologies were chosen (adaptive clinical trial, classical article, clinical study, clinical trial, clinical trial phase I-IV, comparative study, controlled clinical trial, dataset, meta-analysis, pragmatic clinical trial, randomized control trial, twin study, validation study). In PI, searches were restricted to studies in English, humans were selected as species, and quantitative methodologies were chosen (brain imaging, empirical study, quantitative study, interview, experimental replication, clinical case study, follow-up study, longitudinal study, meta-analysis, twin study).

|  | Keywords | Results | |  |  |  |  |  |  |  |
| --- | --- | --- | --- | --- | --- | --- | --- | --- | --- | --- |
|  |  |  |  | After removal due to: | | | | | |  |
|  |  |  |  | Filters added | Duplicates removed | Title/ abstract | Lacking relevant measure | Lack of relevance/ wrong article type | Lack of sex differentiation | Total studies included |
| EQ, SQ & ASD | ((Empathizing quotient) OR (Empathising quotient) OR (empathy quotient) OR (systemizing quotient) OR (systemising quotient) OR (empathizing-systemizing) OR (empathising-systemising)) AND ((Autis*) OR (ASD)) | Pmed  WoS  PI  Other | 215  714  346  1 | 28  657  275 | 758 | 150 | 108 | 96 | 31 | 31 |
