# Supplementary material for: Does the Extreme Male Brain Hypothesis of Autism Apply More to Females Than Males? A Systematic and Meta‐Analytic Approach
Source: Autism Res. 2026 Feb 16;19(4):e70198. doi: 10.1002/aur.70198 (PMC13087839; doi:10.1002/aur.70198)
Supplement: Supplementary file 4 — Table S2: Mean and SD of EQ and SQ scores in autistic and NT males and females, presented as the reported mean divided by the maximum score for the quotient version used multiplied by one hundred for comparison. [file AUR-19-0-s003.docx]

| Supplementary table 2. Mean and SD of EQ and SQ scores in autistic and NT males and females, presented as the reported mean divided by the maximum score for the quotient version used multiplied by one hundred for comparison. | | | | | | | | | | | | | |
| --- | --- | --- | --- | --- | --- | --- | --- | --- | --- | --- | --- | --- | --- |
|  |  | **ASD** | | | **NT** | | | **ASD-NT differences** | | **ASD sex differences** | | **NT sex differences** | |
| STUDy | Sex | n | EQ | SQ | n | EQ | SQ | EQ | SQ | EQ | SQ | EQ | SQ |
|  |  |  | (% max possible score) | |  | (% max possible score) | | Cohen’s d | | Cohen’s d | | Cohen’s d | |
| AUYEUNG ET AL. (2009) | M  F | 219  46 | 25.4 (12.8)  28.5 (11.6) | 49.5 (16.5)  46.6 (16.3) | 581  675 | 64.4 (18.7)  74.4 (16.5) | 46.1 (13.9)  40.4 (13.6) | -2.26  -2.83 | 0.23  0.45 | -0.25 | 0.17 | -0.57 | 0.42 |
| AUYEUNG ET AL. (2012) | M  F | 173  40 | 13.6 (22.1)  19.9 (22.6) | 44.5 (19.9)  37.4 (20.3) | 459  571 | 57.6 (25.3)  66.6 (17.3) | 34.9 (22.7)  27.7 (15.5) | -1.80  -2.64 | 0.44  0.61 | -0.28 | 0.35 | -0.42 | 0.38 |
| BARON-COHEN ET AL. (2014) | M  F | 357  454 | 25.5 (15.5)  33.0 (21.5) | 52.3 (16.2)  47.4 (17.1) | 1344  2562 | 47.5 (17.1)  60.6 (17.1) | 45.4 (14.4)  36.7 (14.1) | -1.31  -1.55 | 0.46  0.73 | -0.39 | 0.29 | -0.77 | 0.61 |
| GREENBERG ET AL. (2018) | M  F | 18188  18460 | 34.6 (9.42)  41.3 (10.1) | 40.4 (9.28)  35.5 (8.74) | 241355  393600 | 44.4 (9.50)  54.0 (9.68) | 33.7 (8.36)  27.3 (7.74) | -1.03  -1.31 | 0.79  1.05 | -0.69 | 0.54 | -1.00 | 0.80 |
| GROEN ET AL. (2015) | M F | 42  - | 33.0 (17.1)  - | 39.9 (13.3)  - | 270  415 | 51.4 (17.5)  64.1 (15.4) | 41.3 (11.9)  32.9 (10.2) | -1.05  - | -0.12  0.74 | - | -2323 | -0.78 | 0.76 |
| GROVE ET AL. (2015) | M  F | 141  170 | 22.6 (13.3)  23.4 (11.8) | 50.9 (16.9)  52.3 (16.8) | 122  110 | 48.1 (16.1)  60.1 (17.4) | 44.7 (15.3)  40.0 (16.3) | -1.74  -2.58 | 0.39  0.74 | -0.06 | -0.08 | -0.72 | 0.30 |
| HENDRIKS ET AL. (2020) | M F | 15  5 | 23.5 (17.0)  37.7 (24.5) | 45.3 (18.9)  38.4 (16.9) | 47  32 | 44.2 (22.9)  51.1 (20.1) | 38.4 (20.8)  40.2 (17.0) | -0.63  -0.33 | 0.39  -0.10 | -0.75 | 0.37 | -0.31 | -0.09 |
| KOSE ET AL. (2025) | M  F | 38  12 | 48.7 (14.9)  53.7 (16.6) | 45.7 (11.6)  45.2 (16.3) | 35  38 | 64.6 (14.4)  74.6 (12.6) | 52.0 (15.3)  56.3 (12.9) | -1.09  -1.53 | -0.46  -0.80 | -0.33 | 0.04 | -0.74 | -0.30 |
| KUNG (2024) | M  F | 29  13 | 23.3 (15.3)  24.6 (13.0) | 35.0 (17.3)  39.0 (18.4) | 205  493 | 40.5 (21.4)  52.0 (19.7) | 36.9 (21.0)  30.7 (17.8) | -0.83  -1.40 | -0.09  0.46 | -0.09 | -0.22 | -0.57 | 0.33 |
| LAI ET AL. (2011) | M  F | 33  29 | 25.1 (13.6)  23.6 (9.50) | 44.6 (22.4)  48.3 (19.5) | -  - | -  - | -  - | -  - | -  - | 0.13 | -0.21 | - | - |
| LEHNHARDT ET AL. (2016) | M F | 69  38 | 15.4 (8.3)  16.5 (7.7) | 41.0 (15.6)  43.7 (13.8) | -  - | -  - | -  - | -  - | -  - | -0.14 | -0.18 | - | - |
| LEPAGE ET AL. (2009) | M  F | 15  8 | 26.8 (9.50)  42.2 (12.2) | -  - | 64  28 | 45.3 (12.4)  53.5 (6.14) | -  - | -1.45  -1.56 | -  - |  | - | -0.75 | - |
| PARK ET AL. (2012) | M  F | 91  20 | 28.9 (11.2)  31.7 (21.5) | 45.4 (10.7)  42.7 (11.4) | 26  25 | 59.3 (19.4)  68.7 (15.5) | 43.8 (13.0)  34.1 (10.9) | -2.26  -2.01 | 0.14  0.77 | -0.14 | 0.04 | -0.54 | 0.80 |
| SHALEV ET AL. (2022) | M  F | 878  1027 | 21.8 (11.9)  24.9 (11.9) | 54.4 (31.3)  51.6 (30.3) | 763  2246 | 49.4 (16.5)  62.0 (16.0) | 45.3 (28.0)  35.6 (26.0) | -1.94  -2.51 | 0.30  0.58 | -0.26 | 0.09 | -0.78 | 0.37 |
| STAUDER ET AL. (2011) | M  F | 16  9 | 30.1 (9.65)  32.4 (9.19) | 52.3 (18.3)  51.1 (17.3) | 16  9 | 55.0 (12.4)  63.4 (12.6) | 49.8 (19.3)  34.0 (9.54) | -2.24  -2.81 | 0.13  1.23 | -0.24 | 0.06 | -0.67 | 0.95 |
| SUCKSMITH ET AL. (2013) | M  F | 161  168 | 21.9 (13.1)  22.8 (11.1) | -  - | 93  94 | 47.1 (16.9)  60.6 (17.6) | - | -1.79  -2.74 | -  - | -0.07 | . | -0.78 | - |
| TAVASSOLI ET AL. (2018) | M  F | 57  11 | 24.8 (13.0)  38.0 (23.0) | 50.5 (16.0)  50.5 (13.6) | 34  29 | 59.1 (21.0)  64.3 (22.4) | 39.5 (13.0)  37.9 (13.0) | -2.09  -1.16 | 0.73  0.96 | -0.88 | 0.00 | -0.24 | 0.12 |
| WAKABAYASHI ET AL. (2007) | M  F | 38  10 | 55.9 (9.58)  59.1 (8.80) | 62.6 (16.2)  73.4 (17.9) | 71  66 | 70.7 (12.0)  83.8 (12.1) | 59.0 (12.9)  34.6 (13.6) | -1.31  -2.11 | 0.25  2.73 | -0.19 | -0.41 | -1.21 | 1.66 |
| WARRIER ET AL. (2020) | M  F | 193398  317891 | 34.6 (23.6)  41.1 (25.3) | 40.3 (23.2)  35.9 (22.1) | 13317  13934 | 44.4 (23.7)  54.0 (24.3) | 33.6 (20.9)  27.2 (19.4) | -0.42  -0.51 | 0.23  0.22 | -0.26 | 0.06 | -0.40 | 0.32 |
| WHEELWRIGHT ET AL. (2006) | M  F | 69  56 | 23.4 (12.3)  23.1 (12.6) | 51.9 (15.3)  50.9 (16.7) | 723  1038 | 48.8 (14.5)  60.0 (14.1) | 40.8 (12.8)  34.5 (12.8) | -1.77  -2.62 | 0.85  1.26 | 0.02 | 0.06 | -0.79 | 0.49 |

References

Auyeung, B., Allison, C., Wheelwright, S., & Baron-Cohen, S. (2012). Brief Report: Development of the Adolescent Empathy and Systemizing Quotients. *Journal of Autism and Developmental Disorders*, *42*(10), 2225–2235.

Auyeung, B., Wheelwright, S., Allison, C., Atkinson, M., Samarawickrema, N., & Baron-Cohen, S. (2009). The Children’s Empathy Quotient and Systemizing Quotient: Sex Differences in Typical Development and in Autism Spectrum Conditions. *Journal of Autism and Developmental Disorders*, *39*(11), 1509–1521.

Baron-Cohen, S., Cassidy, S., Auyeung, B., Allison, C., Achoukhi, M., Robertson, S., Pohl, A., & Lai, M.-C. (2014). Attenuation of Typical Sex Differences in 800 Adults with Autism vs. 3,900 Controls. *PLoS ONE*, *9*(7), e102251.

Greenberg, D. M., Warrier, V., Allison, C., & Baron-Cohen, S. (2018). Testing the Empathizing–Systemizing theory of sex differences and the Extreme Male Brain theory of autism in half a million people. *Proceedings of the National Academy of Sciences*, *115*(48), 12152–12157.

Groen, Y., Fuermaier, A. B. M., Den Heijer, A. E., Tucha, O., & Althaus, M. (2015). The empathy and systemizing quotient: The psychometric properties of the Dutch version and a review of the cross-cultural stability. *Journal of autism and developmental disorders*, *45*(9), 2848-2864.

Grove, R., Baillie, A., Allison, C., Baron-Cohen, S., & Hoekstra, R. A. (2015). Exploring the quantitative nature of empathy, systemising and autistic traits using factor mixture modelling. *British Journal of Psychiatry*, *207*(5), 400–406.

Hendriks, O., Wei, Y., Warrier, V., & Richards, G. (2022). Autistic traits, empathizing–systemizing, and gender diversity. *Archives of sexual behavior*, *51*(4), 2077-2089.

Kose, S., Turer, F., Inal Kaleli, I., Calik Senturk, H. N., Ozuysal Uyar, D. H., & Bildik, T. (2025). The Relationship Between Social Skills and Sensory Profile, Emotion Regulation, and Empathizing/Systemizing in Adolescents on the Autism Spectrum. *Journal of Autism and Developmental Disorders*, *55*(1), 59–75.

Kung, K. T. F. (2024). The Chinese 10-Item Empathy Quotient and Systemising Quotient-Revised: Internal Consistency, Test-Retest Reliability, Known-Groups Validity, and Sex Differences in Autistic and Non-Autistic Adults. *Journal of Autism and Developmental Disorders*. https://doi.org/10.1007/s10803-024-06608-8

Lai, M. C., Lombardo, M. V., Pasco, G., Ruigrok, A. N., Wheelwright, S. J., Sadek, S. A., ... & Baron-Cohen, S. (2011). A behavioral comparison of male and female adults with high functioning autism spectrum conditions. *PloS one*, *6*(6), e20835.

Lehnhardt, F. G., Falter, C. M., Gawronski, A., Pfeiffer, K., Tepest, R., Franklin, J., & Vogeley, K. (2016). Sex-related cognitive profile in autism spectrum disorders diagnosed late in life: implications for the female autistic phenotype. *Journal of autism and developmental disorders*, *46*(1), 139-154.

Lepage, J. F., Lortie, M., Taschereau-Dumouchel, V., & Théoret, H. (2009). Validation of French-Canadian versions of the empathy quotient and autism spectrum quotient. *Canadian Journal of Behavioural Science/Revue canadienne des sciences du comportement*, *41*(4), 272.

Park, S., Cho, S.-C., Cho, I. H., Kim, B.-N., Kim, J.-W., Shin, M.-S., Chung, U.-S., Park, T.-W., Son, J.-W., & Yoo, H. J. (2012). Sex differences in children with autism spectrum disorders compared with their unaffected siblings and typically developing children. *Research in Autism Spectrum Disorders*, *6*(2), 861–870.

Shalev, I., Warrier, V., Greenberg, D. M., Smith, P., Allison, C., Baron‐Cohen, S., Eran, A., & Uzefovsky, F. (2022). Reexamining empathy in autism: Empathic disequilibrium as a novel predictor of autism diagnosis and autistic traits. *Autism Research*, *15*(10), 1917–1928. https://doi.org/10.1002/aur.2794

Stauder, J. E. A., Cornet, L. J. M., & Ponds, R. W. H. M. (2011). The extreme male brain theory and gender role behaviour in persons with an autism spectrum condition. *Research in Autism Spectrum Disorders*, *5*(3), 1209-1214.

Sucksmith, E., Allison, C., Baron-Cohen, S., Chakrabarti, B., & Hoekstra, R. A. (2013). Empathy and emotion recognition in people with autism, first-degree relatives, and controls. *Neuropsychologia*, *51*(1), 98-105.

Tavassoli, T., Miller, L. J., Schoen, S. A., Brout, J. J., Sullivan, J., & Baron-Cohen, S. (2018). Sensory reactivity, empathizing and systemizing in autism spectrum conditions and sensory processing disorder. *Developmental cognitive neuroscience*, *29*, 72-77.

Wakabayashi, A., Baron-Cohen, S., Uchiyama, T., Yoshida, Y., Kuroda, M., & Wheelwright, S. (2007). Empathizing and Systemizing in Adults with and without Autism Spectrum Conditions: Cross-Cultural Stability. *Journal of Autism and Developmental Disorders*, *37*(10), 1823–1832.

Warrier, V., Greenberg, D. M., Weir, E., Buckingham, C., Smith, P., Lai, M.-C., Allison, C., & Baron-Cohen, S. (2020). Elevated rates of autism, other neurodevelopmental and psychiatric diagnoses, and autistic traits in transgender and gender-diverse individuals. *Nature Communications*, *11*(1), 3959.

Wheelwright, S., Baron-Cohen, S., Goldenfeld, N., Delaney, J., Fine, D., Smith, R., Weil, L., & Wakabayashi, A. (2006). Predicting Autism Spectrum Quotient (AQ) from the Systemizing Quotient-Revised (SQ-R) and Empathy Quotient (EQ). *Brain Research*, *1079*(1), 47–56.
