# Supplementary material for: Does the Extreme Male Brain Hypothesis of Autism Apply More to Females Than Males? A Systematic and Meta‐Analytic Approach
Source: Autism Res. 2026 Feb 16;19(4):e70198. doi: 10.1002/aur.70198 (PMC13087839; doi:10.1002/aur.70198)
Supplement: Supplementary file 5 — Table S3: Mean and standard deviations of AQ, EQ and SQ scores in NT males and females, presented as the reported mean divided by the maximum score for the questionnaire version used multiplied by one hundred for comparison. [file AUR-19-0-s001.docx]

| Supplementary table 3. Mean and standard deviations of AQ, EQ and SQ scores in NT males and females, presented as the reported mean divided by the maximum score for the questionnaire version used multiplied by one hundred for comparison. | | | | | | | | | |
| --- | --- | --- | --- | --- | --- | --- | --- | --- | --- |
|  |  | **Males** | | |  |  | **Females** | |  |
| STUDIES | n | AQ | EQ | SQ | n | AQ | EQ | SQ |  |
| Baron-cohen (2014) | 1344 | 40.6 (15.6) | 47.5 (17.1) | 45.4 (14.4) | 2562 | 34.2 (15.2) | 60.6 (17.1) | 36.7 (14.1) |  |
| greenberg et al. (2018) | 241355 | 35.7 (22.7) | 44.4 (9.50) | 33.7 (8.36) | 393600 | 31.6 (22.6) | 54.0 (9.68) | 27.3 (7.74) |  |
| Hendriks et al. (2022) | 18 | 36.2 (20.9) | 44.2 (22.9) | 38.4 (20.8) | 19 | 38.9 (15.4) | 51.1 (20.1) | 40.2 (17.0) |  |
| Hidaka et al. (2023) | 1112 | 45.4 (13.7) | 31.0 (17.2) | 35.0 (18.3) | 1093 | 41.1 (13.9) | 36.5 (17.1) | 21.8 (14.9) |  |
| jankovic-nikolic et al. (2023) | 164 | 29.5 (7.25) | 71.3 (11.0) | 53.8 (12.6) | 187 | 27.7 (8.31) | 73.7 (11.2) | 51.2 (13.6) |  |
| LePage et al. (2009) | 64 | 33.3 (19.1) | 45.3 (12.4) | . | 28 | 26.5 (8.20) | 53.5 (6.14) | . |  |
| morsanyi et al. (2012) | 55 | 31.1 (11.6) | 50.8 (17.1) | 41.8 (13.8) | 70 | 29.0 (8.64) | 59.1 (17.1) | 23.0 (20.7) |  |
| park et al. (2012) | 26 | 31.4 (9.40) | 59.3 (15.5) | 43.8 (10.9) | 25 | 25.9 (9.40) | 68.7 (15.5) | 34.1 (10.9) |  |
| Paul et al. (2021) | 18 | 39.1 (54.3) | 49.2 (15.9) | 49.7 (10.6) | 12 | 37.8 (38.4) | 57.2 (14.1) | 43.3 (11.5) |  |
| procyshyn et al. (2020) | 78 | 39.2 (10.8) | 53.3 (17.0) | 46.0 (15.8) | 94 | 34.6 (11.6) | 58.2 (15.0) | 29.4 (17.8) |  |
| ramachandra et al. (2022) | 218 | 43.9 (11.8) | 47.9 (14.4) | 46.4 (14.7) | 185 | 38.0 (9.74) | 59.2 (14.4) | 37.7 (17.5) |  |
| richards et al. (2022) | 104 | 36.0 (13.3) | 50.6 (14.6) | 40.1 (11.4) | 104 | 30.6 (14.9) | 65.9 (14.6) | 30.7 (14.3) |  |
| Romero-Martinez et al. (2013) | 19 | 29.2 (8.04) | 55.7 (8.07) | 43.4 (10.2) | 23 | 27.4 (9.86) | 56.6 (8.38) | 36.5 (14.0) |  |
| Romero-Martinez et al. (2014) | 18 | 29.0 (10.1) | 55.4 (8.49) | 44.0 (10.7) | 22 | 28.1 (10.1) | 56.7 (8.71) | 36.9 (14.6) |  |
| Rudra et al. (2016) | 21 | 26.4 (5.27) | 86.3 (3.51) | . | 5 | 26.0 (4.60) | 86.3 (1.67) | . |  |
| shalev et al. (2022) | 763 | 39.6 (16.0) | 49.4 (16.0) | 45.3 (26.0) | 2246 | 34.2 (16.6) | 62.0 (16.5) | 35.6 (28.0) |  |
| sindermann et al. (2019) | 304 | 36.0 (11.2) | 45.4 (11.3) | 39.1 (9.57) | 794 | 31.8 (11.1) | 55.5 (11.0) | 32.8 (9.76) |  |
| stagg & vincent et al. (2019) | 28 | 38.0 (14.0) | 47.5 (14.6) | 40.7 (12.0) | 40 | 36.0 (16.0) | 56.3 (10.1) | 30.0 (13.3) |  |
| Voracek & Dressler (2006) | 206 | 40.9 (7.95) | 61.2 (11.4) | 55.0 (17.9) | 217 | 39.3 (7.67) | 67.5 (12.4) | 44.8 (14.7) |  |
| warrier et al. (2020) | 13317 | 48.8 (22.3) | 44.4 (24.3) | 33.6 (19.4) | 13934 | 47.4 (22.8) | 54.0 (23.7) | 27.2 (20.9) |  |
| Wheelwright et al. (2006) | 723 | 34.8 (11.2) | 48.8 (14.1) | 40.8 (12.8) | 1038 | 31.0 (12.4) | 60.0 (14.5) | 34.5 (12.8) |  |
|  |  |  |  |  |  |  |  |  |  |

References

Baron-Cohen, S., Cassidy, S., Auyeung, B., Allison, C., Achoukhi, M., Robertson, S., Pohl, A., & Lai, M.-C. (2014). Attenuation of Typical Sex Differences in 800 Adults with Autism vs. 3,900 Controls. *PLoS ONE*, *9*(7), e102251.

Greenberg, D. M., Warrier, V., Allison, C., & Baron-Cohen, S. (2018). Testing the Empathizing–Systemizing theory of sex differences and the Extreme Male Brain theory of autism in half a million people. *Proceedings of the National Academy of Sciences*, *115*(48), 12152–12157.

Hendriks, O., Wei, Y., Warrier, V., & Richards, G. (2022). Autistic traits, empathizing–systemizing, and gender diversity. *Archives of sexual behavior*, *51*(4), 2077-2089.

Hidaka, S., Gotoh, M., Yamamoto, S., & Wada, M. (2023). Exploring relationships between autistic traits and body temperature, circadian rhythms, and age. *Scientific Reports*, *13*(1), 5888.

Janković-Nikolić, M., Glumbić, N., Mentus-Kandić, T., & Teovanović, P. (2023). The relationship between sex, empathy, systemizing, and autistic traits in primary school children. *Psihologija*, 1-19.

Lepage, J. F., Lortie, M., Taschereau-Dumouchel, V., & Théoret, H. (2009). Validation of French-Canadian versions of the empathy quotient and autism spectrum quotient. *Canadian Journal of Behavioural Science/Revue canadienne des sciences du comportement*, *41*(4), 272.

Morsanyi, K., Primi, C., Handley, S. J., Chiesi, F., & Galli, S. (2012). Are systemizing and autistic traits related to talent and interest in mathematics and engineering? Testing some of the central claims of the empathizing–systemizing theory. *British journal of psychology*, *103*(4), 472-496.

Park, S., Cho, S.-C., Cho, I. H., Kim, B.-N., Kim, J.-W., Shin, M.-S., Chung, U.-S., Park, T.-W., Son, J.-W., & Yoo, H. J. (2012). Sex differences in children with autism spectrum disorders compared with their unaffected siblings and typically developing children. *Research in Autism Spectrum Disorders*, *6*(2), 861–870.

Paul, S., Arora, A., Midha, R., Vu, D., Roy, P. K., & Belmonte, M. K. (2021). Autistic traits and individual brain differences: functional network efficiency reflects attentional and social impairments, structural nodal efficiencies index systemising and theory-of-mind skills. *Molecular autism*, *12*(1), 3.

Procyshyn, T. L., Watson, N. V., & Crespi, B. J. (2020). Experimental empathy induction promotes oxytocin increases and testosterone decreases. *Hormones and behavior*, *117*, 104607.

Ramachandra, V., & Longacre, H. (2022). Unmasking the psychology of recognizing emotions of people wearing masks: The role of empathizing, systemizing, and autistic traits. *Personality and Individual Differences*, *185*, 111249.

Richards, G., Baron-Cohen, S., Warrier, V., Mellor, B., Davies, J., Gee, L., & Galvin, J. (2022). Evidence of partner similarity for autistic traits, systemizing, and theory of mind via facial expressions. *Scientific Reports*, *12*(1), 8451

Romero-Martínez, Á., de Andrés-García, S., Sariñana-González, P., Sanchis-Calatayud, M. V., Roa, J. M., González-Bono, E., & Moya-Albiol, L. (2013). The 2D: 4D ratio and its relationship with other androgenisation parameters in parents of individuals with autism spectrum disorders. *anales de psicología*, *29*(1), 264-271.

Romero-Martínez, A., et al. "High cognitive sensitivity to activational effects of testosterone in parents of offspring with autism spectrum disorders." *Personality and individual differences* 71 (2014): 45-50.

Rudra, A., Ram, J. R., Loucas, T., Belmonte, M. K., & Chakrabarti, B. (2016). Bengali translation and characterisation of four cognitive and trait measures for autism spectrum conditions in India. *Molecular Autism*, *7*(1), 50.

Shalev, I., Warrier, V., Greenberg, D. M., Smith, P., Allison, C., Baron‐Cohen, S., Eran, A., & Uzefovsky, F. (2022). Reexamining empathy in autism: Empathic disequilibrium as a novel predictor of autism diagnosis and autistic traits. *Autism Research*, *15*(10), 1917–1928.

Sindermann, C., Cooper, A., & Montag, C. (2019). Empathy, autistic tendencies, and systemizing tendencies—Relationships between standard self-report measures. *Frontiers in psychiatry*, *10*, 307.

Stagg, S. D., & Vincent, J. (2019). Autistic traits in individuals self-defining as transgender or nonbinary. *European psychiatry*, *61*, 17-22.

Voracek, M., & Dressler, S. G. (2006). Lack of correlation between digit ratio (2D: 4D) and Baron-Cohen’s “Reading the Mind in the Eyes” test, empathy, systemising, and autism-spectrum quotients in a general population sample. *Personality and Individual Differences*, *41*(8), 1481-1491.

Warrier, V., Greenberg, D. M., Weir, E., Buckingham, C., Smith, P., Lai, M.-C., Allison, C., & Baron-Cohen, S. (2020). Elevated rates of autism, other neurodevelopmental and psychiatric diagnoses, and autistic traits in transgender and gender-diverse individuals. *Nature Communications*, *11*(1), 3959.

Wheelwright, S., Baron-Cohen, S., Goldenfeld, N., Delaney, J., Fine, D., Smith, R., Weil, L., & Wakabayashi, A. (2006). Predicting Autism Spectrum Quotient (AQ) from the Systemizing Quotient-Revised (SQ-R) and Empathy Quotient (EQ). *Brain Research*, *1079*(1), 47–56.
